# Atrial Fibrillation as a Rare Complication of Multisystem Inflammatory Syndrome in Children (MIS-C): A Case Report

**DOI:** 10.7759/cureus.42812

**Published:** 2023-08-01

**Authors:** Thaer Abdul Hadi, Carrie Garrison, Theresa Roca

**Affiliations:** 1 Pediatrics, University of Florida, Ascension Sacred Heart, Pensacola, USA; 2 Pediatric Critical Care, University of Florida, Ascension Sacred Heart, Pensacola, USA; 3 Pediatric Cardiology, University of Florida, Ascension Sacred Heart, Pensacola, USA

**Keywords:** arrhythmias, atrial fibrillation, mis-c, multisystem inflammatory syndrome in children, covid-19

## Abstract

Multisystem inflammatory syndrome in children (MIS-C) is known to represent a hyperinflammatory state with multi-organ involvement. Many cardiac manifestations of MIS-C have been described in the literature, including myopericarditis, congestive heart failure, and arrhythmias. We present a 17-year-old male who initially presented in shock with multi-organ failure and met the diagnostic criteria for MIS-C to develop atrial fibrillation later. Atrial fibrillation resolved after initiating treatment for MIS-C. In this paper, we will discuss arrhythmias linked to MIS-C and will describe the course that our patient followed.

## Introduction

Since coronavirus disease 2019 (COVID-19) was first recognized, it has profoundly impacted global health and remains a major public health burden. Numerous efforts were made to further understand it and establish treatment standards. Cardiac manifestations remain of great significance during the acute phase of COVID-19 infection and during multisystem inflammatory syndrome in children (MIS-C). Such cardiac manifestations include myocardial infarction, myocarditis, and different forms of arrhythmias [1,2]. Many reports described the occurrence and frequency of cardiac arrhythmias in the scope of MIS-C, with the most frequent findings being first-degree atrioventricular (AV) block, QTc prolongation, and T wave inversion [3-5]. We present a 17-year-old male treated at our facility for MIS-C with severe shock at the time of presentation, and his treatment course was complicated by atrial fibrillation. We present this case to expand the understanding of EKG abnormalities in MIS-C and the expected course of such manifestations.

## Case presentation

**Figure 1:**
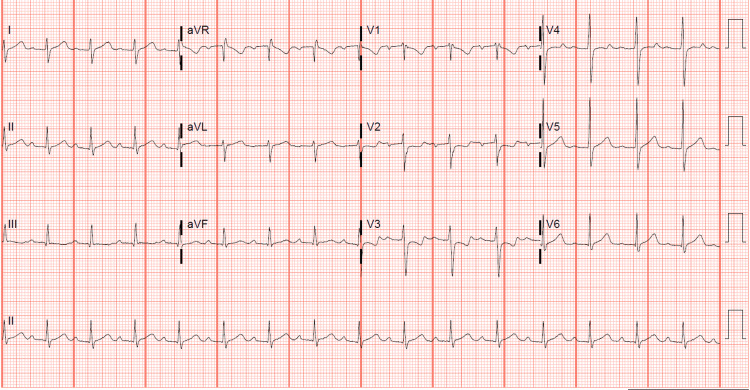
Initial EKG showing first-degree atrioventricular (AV) block.

A 17-year-old healthy male with a recent acute COVID-19 infection with minimal symptoms one month prior to the current presentation presented with a seven-day history of fever. On day six, his symptoms progressed to severe fatigue, injected eyes, visual difficulties with far vision, abdominal pain, and severe diarrhea. He was initially evaluated at an outside emergency department, where he was thought to be in hypovolemic shock with a blood pressure of 66/37 mmHg and a heart rate of 144 bpm. Despite a significant fluid resuscitation, he remained hypotensive, prompting the initiation of an epinephrine drip. Oxygen support by nasal cannula was needed briefly. Based on his clinical presentation and history of a recent acute COVID-19 infection, MIS-C was suspected. MIS-C labs were obtained, which showed: WBC count 12 × 109/L (81% neutrophils and 12% bands), Na 124 mmol/L, creatinine (Cr) 2.14 mg/dL, alanine aminotransferase (ALT) 53 IU/L (normal reference value 11-24), aspartate aminotransferase (AST) 85 IU/L (normal reference value 14-35), ferritin 1713 ng/mL (normal reference value 22-244), troponin 4.4 ng/mL (normal reference value <0.033), brain natriuretic peptide (BNP) 1037 pg/mL (normal reference value 10-100), lipase 215 IU/L (normal reference value 8-78), lactic acid 2.1 mmol/L (normal reference value <2.2), D-dimer 4.8 mcg/mL FEU (normal reference value <0.49), fibrinogen 608 mg/dL (normal reference value 200-400), C-reactive protein (CRP) 28.5 mg/dL (normal reference value <1), erythrocyte sedimentation rate (ESR) 43 mm/hr (normal reference value <10), prothrombin time (PT) 14 seconds, and international normalised ratio (INR) 1.1. Initial EKG showed a first-degree AV block (Figure 1).

CXR was normal. An abdominal ultrasound was obtained due to abdominal pain, and it was negative.

**Figure 2:**
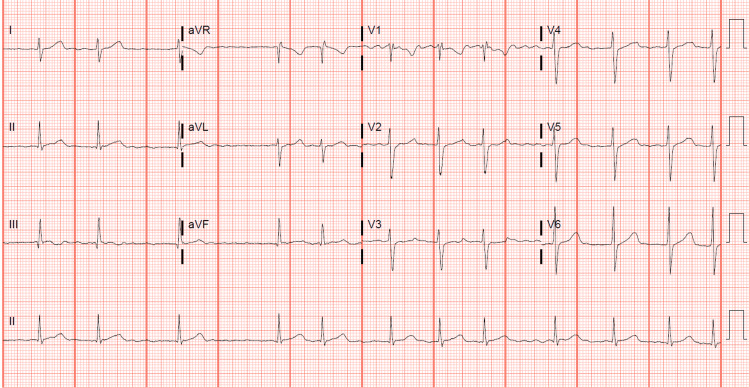
Irregularly irregular ventricular rhythm consistent with atrial fibrillation with a slow ventricular response.

The initial echocardiogram showed an ejection fraction of 40-45%, leading to the conclusion that his shock was also related to decreased cardiac functions. Based on the echocardiogram results, milrinone was initiated in addition to epinephrine. After initial fluid resuscitation and stabilization, he was transferred to our medical facility for further evaluation and management and was admitted to the pediatric intensive care unit. Considering his clinical picture, lab results, and recent history of acute COVID-19 infection, he met the diagnostic criteria for MIS-C based on the latest guidelines from the American College of Rheumatology published in April 2021 [6]. With his presentation of shock and multi-organ involvement, he was started on intravenous immunoglobulin (IVIG) (2 g/kg) and methylprednisolone (2 mg/kg/day). Rocephin was started and continued until the blood cultures ruled out a bacterial blood source for his illness. Repeat labs done at the time of admission to the pediatric intensive care unit showed marked improvement in his electrolytes and kidney functions, but inflammatory and cardiac markers remained elevated. A head CT was obtained for visual changes and was normal. A chest CT to rule out a pulmonary embolism was negative, yet it did show cardiomegaly with a small pericardial effusion, bibasilar peripheral lung consolidations, and small bilateral pleural effusions. Abdominal CT showed thickening of mucosa of the cecum, ascending colon, and rectum. On hospital day two, he was noticed to have an irregular rhythm on the cardiac monitor that was followed by an EKG that showed an irregularly irregular ventricular rhythm. There were no discrete P waves with a chaotic, irregular baseline consistent with atrial fibrillation (Figure 2). Considering that such findings are very unusual for his age group, the rhythm strip was reviewed by pediatric cardiology and EP physicians with consistent readings of it. Although the patient was having atrial fibrillation, his heart rate was within low normal limits, around 70 beats per minute, consistent with a slow ventricular response. The appearance of atrial fibrillation was within 10 hours of starting IVIG, which was infused over 12 hours. The patient described palpitations but no chest pain or dizziness. 

**Figure 3:**
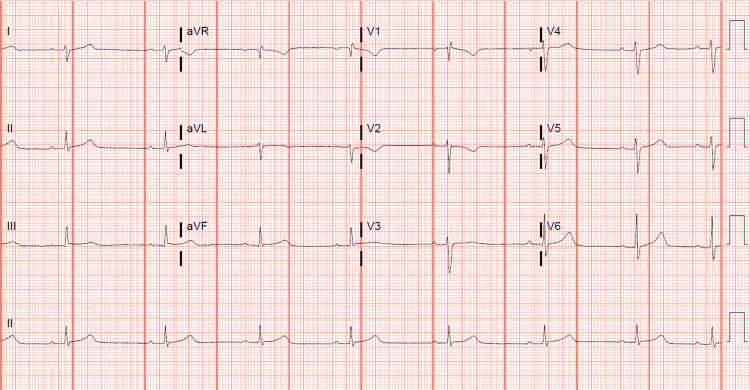
Last EKG before discharge, atrial fibrillation resolved, and reappearance of the first-degree atrioventricular (AV) block with new incomplete right bundle branch block.

The electrophysiology team was consulted and recommended starting the patient on enoxaparin 50 mg twice daily (1 mg/kg twice daily for a dosing weight of 50.5 kg) for thrombosis prophylaxis. No rate control medications were needed, considering the slow ventricular response with a low normal heart rate. Considering the stable status of the patient and the very recent onset of atrial fibrillation in the scope of a known trigger, EP did not recommend chemical or electrical cardioversion as they thought it would likely self-resolve. A repeat echo showed normal cardiac and valvular structures, no atrial thrombi, and an ejection fraction of 52-55%. Cardiac markers remained elevated, with a BNP of 958 pg/mL and a troponin level of 0.235 ng/mL. Milrinone and epinephrine drips were weaned successfully with normalization of his hemodynamics following the IVIG infusion. Within 19 hours after the appearance of atrial fibrillation, it spontaneously resolved with the reappearance of sinus bradycardia with first-degree AV block. The enoxaparin dose was decreased to 40 mg twice daily as a general prophylactic dose, considering the risk of thrombosis with MIS-C in the scope of impaired cardiac functions [6]. The patient showed marked improvement in his symptoms with gradual resolution of elevated inflammatory and cardiac markers following the IVIG infusion. The steroid treatment was continued and tapered over the following two weeks. On the fourth day of his ICU stay, the echo showed persistent improvement. None of the echocardiogram studies showed evidence of coronary artery dilatation. He was discharged the following day after the complete resolution of his symptoms and normalization of his labs, but the persistence of the first-degree AV block (Figure 3).

## Discussion

MIS-C is well known for its pan-systemic involvement and most often manifests as multi-organ damage [6,7]. The heart is not an exception, with many cardiac manifestations described in the literature, including myopericarditis, cardiac tamponade, congestive heart failure, and various arrhythmias [2,8-10].

The pathophysiology of cardiac involvement in MIS-C is yet to be clearly understood but is hypothesized to be a result of severe and widespread cytokine activation with multisystem tissue injury [11], including cardiac and coronary tissue, suggested by cardiac function alterations and elevated cardiac enzymes [9], and proven by cardiac MRI studies [12] and autopsy findings [13]. The injury also involves the electrical conduction system of the heart, resulting in many forms of EKG abnormalities. Many forms of arrhythmias have been reported in MIS-C, with the most common being AV block, especially of the first degree, QTc prolongation, and T wave inversion [3-5]. Regan et al. described the frequency of such arrhythmias in 63 patients with MIS-C. They found that only 33.3% of the patients included in their study did not have ECG changes through their treatment course [5], making it very important to recognize and be aware of such complications.

MIS-C-related arrhythmias being mainly benign and brief might allow the conclusion that such complications stem from the inflammation of the cardiac tissue and electrical conducting system with resolution of such complications once the inflammation is controlled with our known treatment measures of MIS-C. It is of no less importance to describe the expected course of such arrhythmias. Choi et al. presented the outcome of MIS-C-associated AV block among their patients and reported the time for resolution of PR prolongation to occur at a median of three days after the initial appearance of first-degree AV block, with a range of one to five days, suggesting response with the active treatment of MIS-C and resolution of the cardiac muscle inflammation [4]. The case that we are reporting followed a similar course as the atrial fibrillation resolved within one day after IVIG treatment suggesting a good response once inflammation was controlled, although the first-degree AV block persisted at the time of discharge on day five, which might be attributed to MIS-C or to be a baseline EKG finding in this patient. The slow ventricular response that we encountered might suggest AV nodal inflammation leading to a delay in conducting the electrical signals in conjunction with atrial fibrillation.

## Conclusions

MIS-C was linked to many cardiac complications, including myopericarditis, cardiac tamponade, congestive heart failure, and arrhythmias, with the most reported being AV blocks, QTc prolongation, and T wave inversion. Such manifestations during MIS-C are likely a result of cytokine activation with cardiac, coronary, and conductive tissue inflammation. The patient we described presented with MIS-C and acutely developed atrial fibrillation with a slow ventricular response that resolved within a day after starting IVIG, concluding that atrial fibrillation was likely a direct result of cardiac and conductive tissue inflammation and thus subsided once anti-inflammatory medications were started. 
